# Vectorized rooftop area data for 90 cities in China

**DOI:** 10.1038/s41597-022-01168-x

**Published:** 2022-03-02

**Authors:** Zhixin Zhang, Zhen Qian, Teng Zhong, Min Chen, Kai Zhang, Yue Yang, Rui Zhu, Fan Zhang, Haoran Zhang, Fangzhuo Zhou, Jianing Yu, Bingyue Zhang, Guonian Lü, Jinyue Yan

**Affiliations:** 1 Key Laboratory of Virtual Geographic Environment (Ministry of Education of PRC), Nanjing Normal University, Nanjing, 210023 China; 2 State Key Laboratory Cultivation Base of Geographical Environment Evolution, Nanjing, 210023 China; 3 Jiangsu Center for Collaborative Innovation in Geographical Information Resource Development and Application, Nanjing, 210023 China; 4 Jiangsu Provincial Key Laboratory for NSLSCS, School of Mathematical Science, Nanjing Normal University, Nanjing, 210023 China; 5 Department of Land Surveying and Geo-Informatics, The Hong Kong Polytechnic University, Kowloon, Hong Kong China; 6 Senseable City Lab, Massachusetts Institute of Technology, Cambridge, MA 02139 USA; 7 Center for Spatial Information Science, The University of Tokyo, 5-1-5 Kashiwanoha, Kashiwa-shi, Chiba, 277-8568 Japan; 8 LocationMind Inc, 3-5-2 Iwamotocho, Chiyoda-ku, Tokyo, 101-0032 Japan; 9 Future Energy Center, Malardalen University, 72123 Vasteras, Sweden; 10 Department of Chemical Engineering, KTH Royal Institute of Technology, Stockholm, 10044 Sweden

**Keywords:** Geography, Environmental sciences

## Abstract

Reliable information on building rooftops is crucial for utilizing limited urban space effectively. In recent decades, the demand for accurate and up-to-date data on the areas of rooftops on a large-scale is increasing. However, obtaining these data is challenging due to the limited capability of conventional computer vision methods and the high cost of 3D modeling involving aerial photogrammetry. In this study, a geospatial artificial intelligence framework is presented to obtain data for rooftops using high-resolution open-access remote sensing imagery. This framework is used to generate vectorized data for rooftops in 90 cities in China. The data was validated on test samples of 180 km^2^ across different regions with spatial resolution, overall accuracy, and F1 score of 1 m, 97.95%, and 83.11%, respectively. In addition, the generated rooftop area conforms to the urban morphological characteristics and reflects urbanization level. These results demonstrate that the generated dataset can be used for data support and decision-making that can facilitate sustainable urban development effectively.

## Abstract (summary)

**Table:** 

Measurement(s)	building rooftop area
Technology Type(s)	computational modeling technique
Sample Characteristic - Environment	city
Sample Characteristic - Location	China

## Background & Summary

Rooftops of buildings have been intensively studied in fields such as sustainable urban development, building energy modeling, and urban planning and design in recent decades^1–3^. Owing to urbanization associated with the digital age, reliable information on rooftops is in increasing demand^4–6^. The rapid access to accurate rooftop information is important for the evaluation of urban and rural development trends. These trends are useful for formulating development strategies and protecting urban and rural ecosystems^7–9^. However, data on rooftop areas are unavailable in many developing countries because of resource constraints. Therefore, methods suitable for generating reliable data on rooftop areas of buildings at low cost are urgently needed^10–12^.

The automatic extraction of rooftop area data is gaining popularity in diverse fields, and studies involving varied data sources exist^13^. Three-dimensional (3D) spatial data, such as the Digital Surface Model (DSM) and Light Detection and Ranging (LiDAR), are exploited for reconstructing buildings, which includes the rooftop area representation and geometric modeling^13–16^. However, the costs of acquiring 3D spatial data and of constructing the associated 3D models are costly, especially at the city scale. Due to the development of image processing algorithms, such as the edge detection and image segmentation, rooftops data can be extracted from high-resolution remote sensing imagery^17,18^. Conventional image process techniques, however, involve complex empirical rules and threshold settings, and thus, exhibit limitations when applied to high-resolution remote sensing imagery in large-scale^14^.

Open-access data from public service providers, such as Google Earth, Baidu Map, and OpenStreetMap, provide opportunities for the acquisition of urban information associated with broad coverage, fast updating speeds, and low cost^19–21^. However, although open-access data, for example, from a Google Earth Satellite (GES) image are valuable for obtaining information on cities, conventional processing methods hardly discover in-depth semantic information and lack flexibility when examining large data involving complex features^22^.

In recent years, deep learning methods have been employed for efficient feature learning and urban information acquisition^23,24^. In fact, deep learning-based image semantic segmentation methods have been applied for the extraction of rooftops data^25,26^. Nevertheless, regarding the optimization of rooftop data extraction applications, the data acquisition process requires the incorporation of geographic information^27,28^. In addition, public rooftop area datasets that are suitable for use as training dataset in machine learning are scant^29^. Further, naive deep learning models based on unbalanced and insufficient training samples exhibit unsatisfactory performances^30^. Therefore, a robust high-performance rooftop extraction model remains elusive in China.

**Fig. 1:**
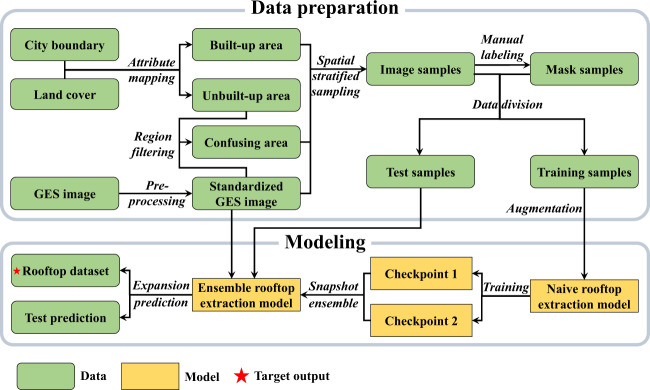
The framework of obtaining rooftop area data in China.

In the present study, the main objective is to extract accurate rooftop areas in China using high-resolution open-access remote sensing imagery based on a geospatial artificial intelligence (GeoAI) framework. The principal components of this framework are illustrated in Fig. 1. The following steps were employed for generating the rooftop area dataset: (1) data preparation through spatial stratified sampling involving geospatial prior knowledge and data processing pipeline to augment the representativeness and number of samples; (2) creation of a deep learning segmentation model, which is based on an ensemble learning strategy and an improved prediction method to improve the rooftop extraction performance.

Based on the framework, we developed a national-scale vectorized rooftop area dataset involving 90 cities in China. The data was validated on test samples of 180 km^2^ across different regions with spatial resolution, overall accuracy, and F1 score of 1 m, 97.95%, and 83.11%, respectively.

## Methods

### Data collection

#### GES imagery

In the present study, open-access GES imagery served as the primary data source (Table 1). GES imagery is advantageous because of its high resolution and wide coverage. These images were downloaded in October 2020 using Python scripts in the open map service application program interface (Google Earth API) provided by Google. The spatial resolution of GES imagery varies according to its image level. The spatial resolution of the 18-level GES imagery was approximately 0.6 m/pixel in most developed countries, and this adequately displayed the geometry and structure of different rooftops. However, because the GES images originated from multiple sensors, differences in their performance induced variations in the resolution from region to region. For example, in China, images of major cities are usually obtained from the WorldView, and QuickBird satellites, and these involve an original resolution within 1 m. In contrast, images of remote areas are acquired from the SPOT series satellites, and the original resolution of which is within 5 m.

**Table 1:** Data type, provided information, and the source used for accessing data involved in the present study.

Data	Information	Source
GES imagery	high spatial resolution satellite imagery data	https://www.google.com/earth
FROM-GLC30	30-m spatial resolution global land cover data	http://data.ess.tsinghua.edu.cn

#### FROM-GLC30

The spatial stratified sampling standard was based on a priori knowledge of the urban land cover from the global 30 m resolution land cover data (FROM-GLC30) created in 2017 (Table 1). These data include the following types: cultivated land, woodland, grassland, shrubland, wetland, waterbody, tundra, artificial surface, bare land, glacier, and permafrost. The overall accuracy of the FROM-GLC30 data is 72.43%^31^, which is based on a global all-season validation sample set from more than 36,000 locations.

### Site selection

**Fig. 2:**
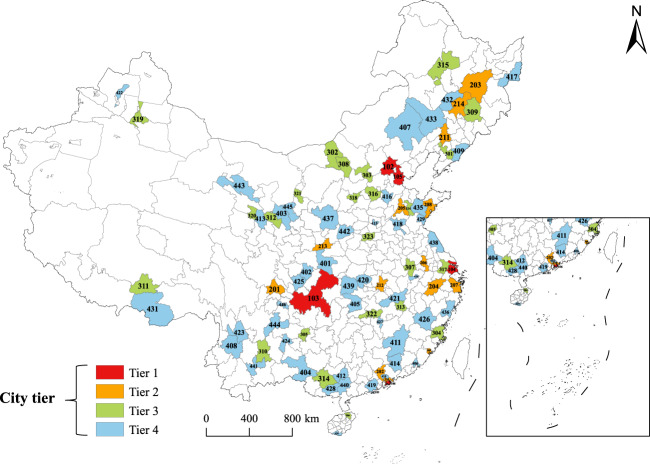
The 90 selected cities in China organized in a hierarchical of four tiers.

In the present study, we selected 90 cities in China (Table 2), and these were partitioned into four tiers based on the city administration hierarchy established by the government in China. Tier 1 involved municipalities with a central administration and regions linked to a special administration in China. Tier 2 comprised mainly sub-provincial cities, while Tier 3 involved provincial capitals and major prefecture-level cities. Tier 4 contained ordinary prefecture-level cities, and the locations of these 90 cities are displayed in Fig. 2. These cities involve all provincial capitals and major administrative levels in China, and their distribution covers different climate regions, and thus, highlight the economics, political, and geographic adequacy. In terms of population, the 90 selected cities cover about 40% of the entire China.

**Table 2:** Data for the 90 cities in China involved in the present study.

Tier 1 (Count: 6)		Tier 2 (Count: 14)		Tier 3 (Count: 24)		Tier 4 (Count: 46)			
Name	Code	Name	Code	Name	Code	Name	Code	Name	Code
Macao	101	Chengdu	201	Anshan	301	Ankang	401	Bazhong	402
Beijing	102	Guangzhou	202	Baotou	302	Baiyin	403	Baise	404
Chongqing	103	Harbin	203	Datong	303	Changde	405	Chaozhou	406
Shanghai	104	Hangzhou	204	Fuzhou	304	Chifeng	407	Dali	408
Tianjin	105	Jinan	205	Guiyang	305	Datong	409	Dongguan	410
Hong Kong	106	Nanjing	206	Haikou	306	Ganzhou	411	Guigang	412
		Ningbo	207	Hefei	307	Haidong	413	Heyuan	414
		Qingdao	208	Hohhot	308	Hebi	415	Hengshui	416
		Xiamen	209	Jilin	309	Jixi	417	Jining	418
		Shenzhen	210	Kunming	310	Jiangmen	419	Jingmen	420
		Shenyang	211	Lhasa	311	Jiujiang	421	Karamay	422
		Wuhan	212	Lanzhou	312	Lijiang	423	Liupanshui	424
		Xi’an	213	Nanchang	313	Nanchong	425	Nanping	426
		Changchun	214	Nanning	314	Pingxiang	427	Qinzhou	428
				Qiqihar	315	Rizhao	429	Sanya	430
				Shijiazhuang	316	Shannan	431	Songyuan	432
				Suzhou	317	Tongliao	433	Tongling	434
				Taiyuan	318	Weifang	435	Wenzhou	436
				Urumqi	319	Yan’an	437	Yancheng	438
				Xining	320	Yichang	439	Yulin (Guangxi Province)	440
				Yinchuan	321	Yuxi	441	Yuncheng	442
				Changsha	322	Zhangye	443	Zhaotong	444
				Zhengzhou	323	Zhongwei	445	Zigong	446
				Zibo	324

Information about the characteristics of the 90 cities in each tier is presented in Table 3 (collated from national statistics^32,33^, no data available for Hong Kong and Macau). The administrative center bias in resource allocation makes cities with higher administrative rank more advantageous in terms of expansion. Therefore, the average size of population and built district shows an increasing trend from Tier 4 to Tier 1. Cities in Tier 1 and Tier 2 are mostly the economic centers of provinces, with developed economies and high urbanization rates, and usually show a multi-core urban morphology. Cities in Tier 3 and Tier 4, on the other hand, usually have a single-core urban morphology. In addition, the shape of cities in each tier generally varies depending on the topography. Cities in the plains tend to have a clumped shape, while cities along rivers and valleys tend to have a striped shape.

**Table 3:** Characteristics information of the 90 cities in different tiers.

Characteristics		Tier 1	Tier 2	Tier 3	Tier 4
Area of administrative district (km^2^)	MIN	6,340.50	1,516.00	2,315.00	1,918.00
	AVE	29,271.87	13,452.85	14,799.38	19,749.02
	MAX	82,370.00	53,186.00	44,287.00	90,064.00
Area of built district (km^2^)	MIN	1,151.05	354.79	87.27	10.80
	AVE	1,343.34	714.08	296.36	131.11
	MAX	1,515.41	1,324.17	580.75	1,194.31
Permanent population (Ten thousand)	MIN	1,386.60	516.40	86.79	35.40
	AVE	2,317.11	1,183.23	587.13	362.64
	MAX	3,205.42	2,093.78	1,274.83	1,046.66

### Data preparation

#### GES imagery preprocessing

The quality of a GES image varies based on the imaging sensor, imaging time, and environmental factors (e.g., atmospheric condition and climate), all of which affect the model training and generalization. Therefore, standardization procedure for GES imagery is necessary, and in the present study, the Gamma Correction algorithm^34^ and Contrast Limited Adaptive Histogram Equalization algorithm^35^ were used to resolve brightness and sharpness issues.

#### Spatial stratified sampling strategy

The study area involves several land cover types, and the proportion of unbuilt areas including water, cultivated land, and forest, exceeds that of built-up areas. Therefore, regular random sampling will create the imbalanced category problem, in which the proportion of negative samples (non-rooftop samples) significantly surpasses that of positive samples (rooftop samples). Therefore, the priori knowledge of the urban land cover was utilized to partition the study area into built-up and unbuilt areas based on the FROM-GLC30 data. The built-up area contains mainly artificial surfaces, which easily yield positive samples. In contrast, the unbuilt area comprises water bodies, wetlands, grasslands, bare lands, cultivated lands, shrublands, and forests, which commonly provide negative samples.

However, based on empirical evidence from previous studies in unbuilt areas, we find farmlands, bare lands, and intersections of different land cover types can be misclassified by the rooftop extraction model. Therefore, in the present study, the unbuilt areas were manually filtered to determine confusing areas, and these areas supplied confusing negative samples.

**Fig. 3:**
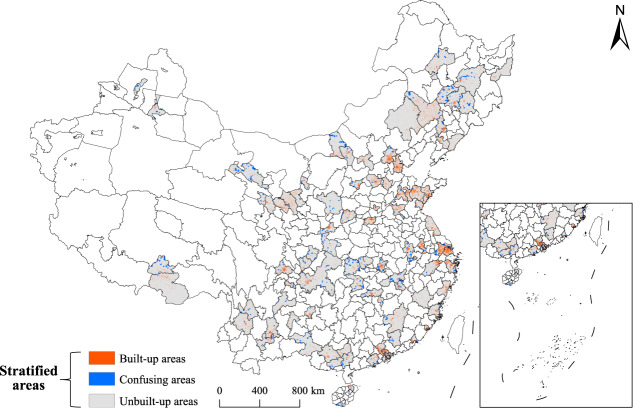
Redundant information of stratified sampling in the study area.

Therefore, to obtain representative and balanced positive and negative samples, spatial stratified sampling was employed in the sample acquisition. The results of stratified sampling in the built-up and confusing areas are displayed in Fig. 3, and this approach was used to produce patches from the GES images.

#### Sample processing and division

In the present study, sample images were correspondingly labeled positive and negative manually. Patches of the image samples and the corresponding masks were then divided into training samples (served as input for the rooftop extraction model training) and test samples (served as ground truth for the validation of rooftop area dataset).

During the rooftop extraction model training task, the built-up area covered by the image samples and their corresponding masks was 200 km^2^, while the confusing area was 160 km^2^, and thus, the total training samples covering 360 km^2^. According to previous deep learning studies, higher training data volume produce more robust models. However, in practice, available data are usually limited. To resolve this limitation, data augmentation without changing the labeled categories was conducted, thereby enhancing the generalization potential of the model. Data augmentation operations used in the present study included the following: random cropping, image rotation, image flipping, image blurring, and noise addition.

### Modelling

#### Naive rooftop extraction model

Rooftop areas of cities were extracted using the DeepLabV3+ model and GES images. The DeepLabV3+ is an open-source image semantic segmentation model that was launched by the Google R&D team^36^. In GES images, the rooftop areas exhibit varied sizes and shapes because of differences in architectural styles and dimensions^37^. Rooftop edges are sometimes difficult to accurately identify because the GES image quality is affected by weather conditions^38^. DeepLabV3 + can perceive features of different scales, thereby improving the recognition accuracy for multifarious rooftops. In addition, the DeepLabV3+ enables transformation of the feature map into a constant resolution map based on the encoder–decoder structure, and this resolves the blurred edges of the rooftop area extraction issue^39^.

In the present study, the cross-entropy^40^ and dice loss functions^41^ were integrated to generate a composite loss function that can simultaneously handle the imbalance categories of samples problem and accelerate the convergence of training. These functions are expressed as follows:

1 $$L\left({p}_{i},{p}_{i}\ast \right)=\alpha \ast {L}_{dice}\left({p}_{i},{p}_{i}\ast \right)+\beta \ast {L}_{bce}\left({p}_{i},{p}_{i}\ast \right)$$

where *p*_*i*_ denotes the predicted value of the *i*^*th*^ sample, ${p}_{i}^{\ast }$ represents the ground truth value of the ith sample, *L*_*dice*_(·) is the dice loss function, *L*_*bce*_(·) stands for the cross-entropy loss function, and *α* and *β* are weight coefficients of the loss function, with corresponding values of 0.2 and 0.8 in the present study.

#### Expansion prediction

To extract rooftop areas, standardized GES images served as input for the ensemble model. Considering that original GES images were significantly larger than the required dimension for model input, cropping into smaller patches was necessary for the prediction. However, this creates an uneven transition or stitched problem at the splicing gap of the prediction result of cropped smaller images^42^. The expansion prediction techniques are suitable for eliminating this uneven transition at the splicing gap.

**Fig. 4:**
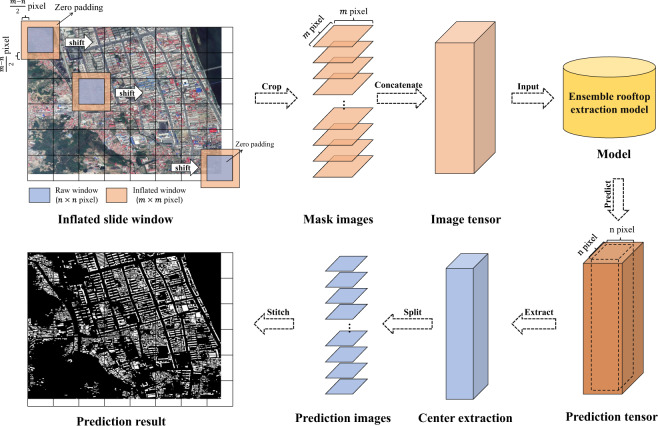
Illustration of steps involved in the expansion prediction method.

Steps implemented in the expansion prediction (Fig. 4) include the following: (1) An n × n sliding window characterized by a step size of n was created. During movement, this window expanded to m × m, and the original remote sensing image was then cropped into small patches. (2) The cropped images were concatenated into a tensor. (3) The ensemble rooftop extraction model was then used to predict the tensor obtained in step 2. (4) The central portion of each n x n tensor element was extracted and split into patches. (5) Patches obtained in step 4 were stitched to produce a large predicted image, which was then cropped based on the original GES image.

#### Model training

Overall network parameters were fine-tuned using the MS COCO dataset^43^ and pre-trained Xception backbone network^44^, while the AdamW optimizer^45^ and Cosine Annealing Warm Restarts algorithm^46^ were employed for rapid convergence of the network. Four Tesla V100 GPUs were used to train the model using the mixed accuracy method, and the development was based on the Pytorch framework^47^. The hyperparameter settings are presented in Table 4.

**Table 4:** Summary of data for hyperparameters utilized in the present study.

Hyperparameter	Value
Learning rate	0.02
Weight decay	0.0005
*T*_0_	2
*T*_*mult*_	2
Output stride	16
Size of input image	384

The parameter *T*_0_ refers to number of iterations in the first restart, while *T*_*mult*_ denotes the increase factor in the Cosine Annealing Warm Restarts.

After the model reaches convergence, rooftop areas were predicted using the snapshot^48^ based on union ensemble rule and expansion prediction techniques, the union ensemble rule is defined in Eq. 2. Considering the trade-off between the quality and speed of prediction, two local optimal models (in this work, checkpoints of the 29^th^ and 55^th^ epochs were chosen) were integrated into the ensemble rooftop extraction model. In addition, the TensorRT framework was used to accelerate the model calculation based on the FP16 accuracy. Accordingly, the prediction speed was increased approximately 5-fold without a significant loss in the accuracy, thereby reducing the prediction time from 100 to 20 days.

2 $${P}_{union-ensemble}=\mathop{\bigcup }\limits_{i=1}^{N}{P}_{i}$$

where *P*_*i*_ denotes the matrix of the prediction category, ${P}_{union-ensemble}$ represents the result of the union integration, and *N* is the number of sub models.

## Data Records

### Data file path

The rooftop area dataset will be updated regularly corresponding to urbanization process of different cities in China. The dataset, metadata, and detailed documentation are freely available for all users at National Tibetan Plateau Data Center (10.11888/Geogra.tpdc.271702)^49^.

### Data file format

The rooftop area dataset is arranged on the tier of cities, where rooftop area data of each city is ESRI Shapefile format^50^, which is composed of .shx, .shp, .prj, .dbf, and .cpg files. The dataset is divided into the original version and the simplified version. The original version is converted by prediction results from model directly, and on which simplified version is obtained by using the Douglas–Peucker algorithm^51^. The total sizes of two version dataset are 118 GB and 21.3 GB without compression.

### File structure

The rooftop area data of each city contains three fields (area, X and Y), as presented in Table 5. All area fields are double float format, in square meters, calculated in CGCS 2000 Albers geographic coordinates. All X and Y fields are double float format, in decimal degrees, calculated in WGS 1984 Web Mercator Auxiliary Sphere geographic coordinates.

**Table 5:** Field description for rooftop area dataset.

Field	Format	Definition	Unit	Geographic reference
Area	double float	Area of each rooftop feature	Square meter	CGCS 2000 Albers
X	double float	Longitude of the central point of each rooftop feature	Decimal degree	WGS 1984 Web Mercator Auxiliary Sphere
Y	double float	Latitude of the central point of each rooftop feature	Decimal degree	WGS 1984 Web Mercator Auxiliary Sphere

## Technical Validation

### Sampling design

Based on the spatial stratified sampling method proposed in this study, the test samples used for the validation of the rooftop area dataset are obtained from GES images and manually labeled with the ground truth through visual interpretation. To better reflect the quality of the rooftop extraction results in each city tier, four tiers of 45 km^2^ were created, and these produced a test dataset covering 180 km^2^, in which the built-up and the confusing areas covered by the image samples and their corresponding masks were 100 and 80 km^2^, respectively.

### Analysis design

Qualitative and quantitative evaluation criteria were utilized to validate the rooftop area dataset generated. Regarding the qualitative evaluation, morphological and topological characteristics of ground truth and extracted rooftop area data for various city tiers were compared. Conversely, for the quantitative evaluation, a testing dataset covering 180 km^2^ comprising four city tiers of 45 km^2^ was employed. In the present study, indicators calculated based on the confusion matrix^52^ include accuracy, precision, recall, and F1 score were used. The closer the values of these indicators are to 100%, the higher the quality of the rooftop extraction. It should be mentioned that precision is equivalent to user accuracy, which is a measure of exactness, and recall is equivalent to producer accuracy, which is a measure of completeness. Precision and recall can also be communicated in terms of error, as either commission error (1-precision) or omission error (1-recall). In particular, the F1 score, which is a weighted average of the recall and precision, is an important indicator for comprehensive evaluation of rooftop extraction results. These data and indicators were then used to evaluate the relationship between the rooftop extraction results and the ground truth^53^.

### Validation results

The validation results for various city tiers are presented in Table 6. The overall accuracy for all city tiers is 97.95%, while the F1 score is 83.11%. However, the overall quality of the rooftop area dataset for high-tier cities is better than that of the lower-tier cities. Based on empirical exploration, we realized that the GES image quality for various cities varied according to the imaging sensor, imaging time, and environmental factors such as the atmospheric condition and climate. These image quality differences affect the model training and generalization. The quality of GES images for high-tier cities is better, so the results of the model extraction are generally better, which explains the quality difference between the rooftop area dataset for different city tiers. Therefore, in using this dataset for other applications, an evaluation of the impact of regional differences in quality on specific applications is necessary.

**Table 6:** Summarized data from the evaluation of rooftop extraction results associated with different city tiers.

City tier	Accuracy (%)	F1 score (%)	Producer accuracy/Recall (%)	User accuracy/Precision (%)	Omission error (%)	Commission error (%)
Tier 1	98.17	85.58	83.70	87.54	16.30	12.46
Tier 2	97.60	83.57	79.65	87.89	20.35	12.11
Tier 3	98.16	83.45	78.43	89.17	21.57	10.83
Tier 4	97.95	82.13	78.21	86.46	21.79	13.54
Overall	97.95	83.11	78.96	87.77	21.04	12.23

**Fig. 5:**
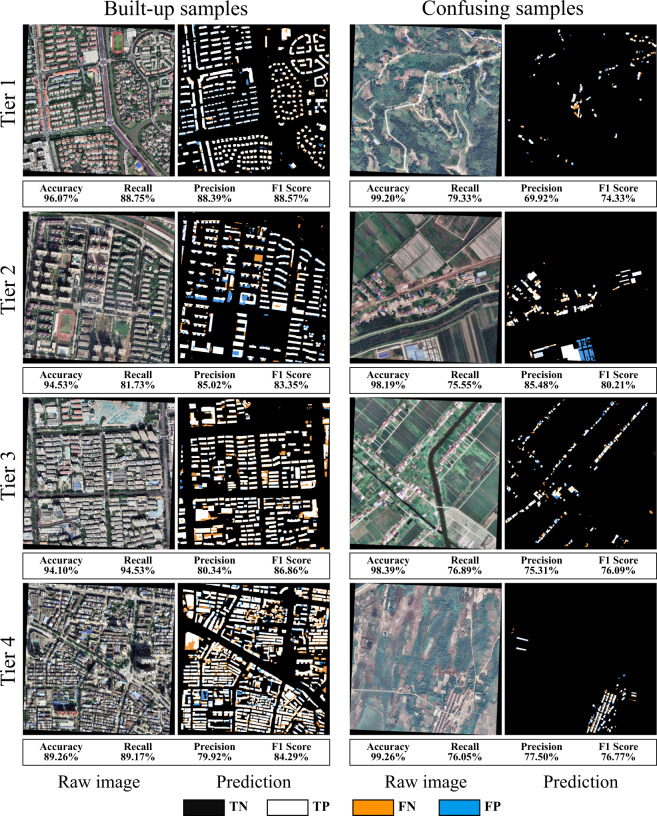
Images highlighting the evaluation of the rooftop area dataset for different city tiers and sampling areas, using different colors to visualize omission and commission errors.

In addition, we validated the quality of the rooftop area dataset for different city tiers using two types of sampling areas, as shown in Fig. 5. Each submap corresponds to a ground extent of 1 km^2^ and the indicators for evaluating the rooftop extraction results are given below. In addition, we visualized the elements TN (True Negative), TP (True Positive), FN (False Negative) and FP (False Positive) in the confusion matrix. In this way the commission and omission errors in the extraction results can be clearly indicated by FP (in blue) and FN (in orange), respectively. It can be seen that the rooftop extraction in the built-up area is better than that in the confusing area, which explains why we use a stratified sampling strategy when collecting samples. In general, the extraction results adequately delineate characteristics of the rooftops, and these are consistent with the GES images. Our dataset also comprises small, sparsely distributed, and irregularly structured rooftops in confusing area, which highlights that the rooftop area dataset generated by the proposed framework has good performance in finding details and effectively avoids the interference of complex background information.

**Fig. 6:**
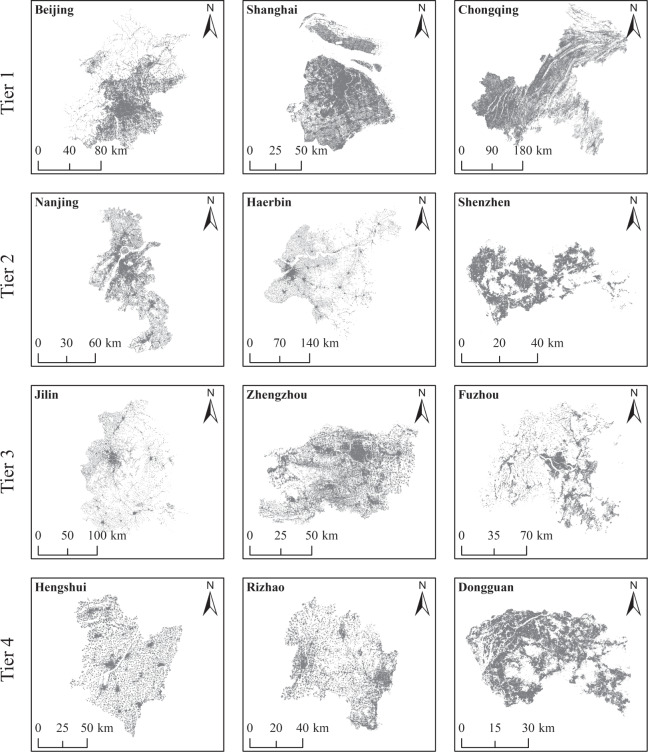
Images highlighting the integrity of the rooftop area dataset for different city tiers, displaying results in both urban and rural space within city boundaries.

Examples of rooftop area dataset for different city tiers are shown in Fig. 6. The extracted rooftop areas exhibit clustering, and this is consistent with the spatial morphology and city boundaries. Therefore, the spatial distribution of rooftop areas is supportive for understanding the urbanization level and urban planning needs of cities. For example, Shanghai is a high-density mega-city in China, and its frank and vast plains and numerous waterways and ports provide advantages for the establishment of its polycentric urban spatial system. The concept of intensive development has also resulted in a more compact urban space in Shanghai. However, the eastern and northern parts of Harbin are mountainous and hilly, and the expansion of the city is limited by natural conditions, so the urban space is loose and the urban area is mainly distributed in the central and western plains.

**Fig. 7:**
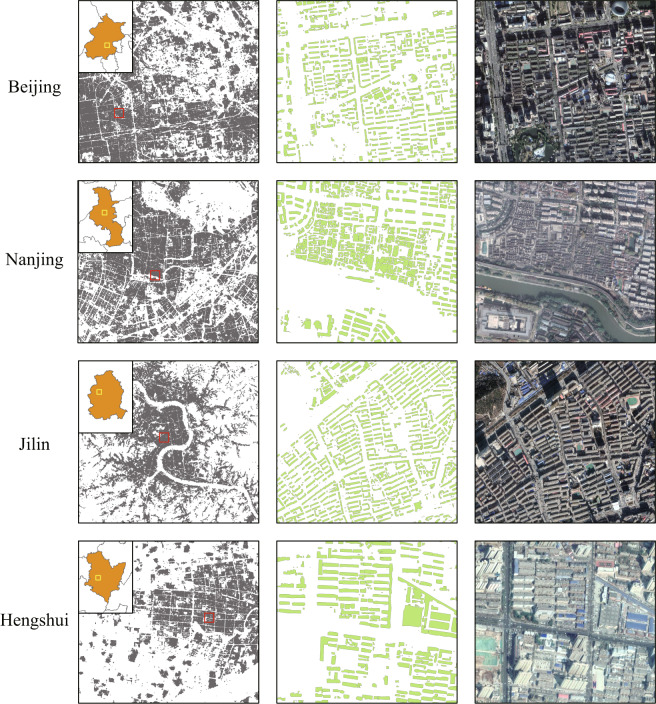
Images for extracted rooftop areas in different cities, indicating the position offset on buildings of different heights.

Specific details on the rooftop area dataset for different city tiers are displayed in Fig. 7 using Beijing, Nanjing, Jilin, and Hengshui as examples. Compared with the GES images, the rooftop extraction results display significant details for various cities, with rooftops and complex backgrounds effectively distinguished. Moreover, dense and sparse spatial distribution areas are accurately extracted. In fact, the extraction results exhibit no sign of the stitched problem because of the application of the expansion prediction.

Meanwhile, Fig. 7 reveals that the GES image used in the present study was not adequately orthorectified, and this partially accounts for the offset in positions between the extracted rooftop area and the ground truth. This offset in positions is significant (approximately 20 m based on empirical survey) for a few high-rise buildings, but it is negligible for low- and medium-rise buildings. Therefore, if the rooftop area data are used for a major city- or country-scale estimation, such position offsets will minimally impact the results. Nevertheless, the rooftop extraction results obtained in the present study provide high-quality details suitable for supporting many architecture-oriented applications.

The empirical evidence of our study in 90 Chinese cities shows that the proposed method can be quickly generalized at a large scale and shows strong robustness in regions with different characteristics. When it is to be extended to regions outside of China, we suggest fine-tuning the existing model by adding new feature samples, to enhance its applicability in the target region.

## Data Availability

The procedure of spatial sampling is executed in the ArcGIS Pro platform. The code of the deep learning model is available at https://github.com/ChanceQZ/RoofTopSegmatation. The program is described by Python3, packages of which are Pytroch, Numpy, and OpenCV mainly.
